# EV-microRNA signatures in pregnant women with idiopathic recurrent pregnancy loss: deciphering microRNAome pathway networks at feto-maternal interface

**DOI:** 10.3389/fimmu.2025.1578738

**Published:** 2025-05-12

**Authors:** Chitra Bhardwaj, Minakshi Rohilla, Seema Chopra, Anupriya Kaur, Inusha Panigrahi, Priyanka Srivastava

**Affiliations:** ^1^ Genetic Metabolic Unit, Department of Pediatrics, Advanced Pediatrics Centre, Postgraduate Institute of Medical Education & Research, Chandigarh, India; ^2^ Department of Obstetrics & Gynaecology, Postgraduate Institute of Medical Education & Research, Chandigarh, India

**Keywords:** recurrent pregnancy loss, extracellular vesicles, miRNA, feto-maternal interface, biomarker, small RNA sequencing

## Abstract

**Background:**

Despite extensive research in the past decade, the exact pathogenesis of recurrent pregnancy loss (RPL) remains unknown. At the time of pregnancy, human placenta releases microRNAs (miRNAs) enclosed in extracellular vesicles (EVs), which enter into maternal circulation and play an important role at feto-maternal interface to sustain a successful pregnancy. Aberrant expression of these miRNAs often results in adverse pregnancy complications. Therefore, studying the expression of these EV-miRNAs in maternal circulation could provide insights into the pathogenesis of RPL.

**Methods:**

The present study included idiopathic currently pregnant (<22 weeks of gestation) RPL women (n=10) and gestational-age-matched healthy pregnant women as control (n=5). EVs were isolated from plasma samples and characterized for their morphology and cell-surface marker. Total RNA was isolated and subjected to miRNA sequencing on Illumina NovaSeq 6000 platform. Differentially expressed (DE) miRNAs were identified using DESeq package. Target prediction and pathway analysis were done using TargetScan, miRDB, miRTarBase, and DIANA-miRPath v3.0 online tool. Protein–protein interaction was done using STRING, and hub genes were identified using Cytoscape software.

**Results:**

miRNA sequencing revealed 66 (44 known and 22 novel) significantly DE miRNAs between RPL and healthy pregnant women. Among these, 37 were downregulated and 29 were upregulated, log2|FC| ≥ 1. Network-based analysis showed highest degree for nine miRNAs (hsa-miR-155-5p, hsa-miR-26a-5p, hsa-miR-204-5p, hsa-miR-140-5p, hsa-miR-139-5p, hsa-let-7e-5p, hsa-miR-149-5p, hsa-miR-374a-5p, and hsa-miR-190a-5p). Gene Ontology (GO) and KEGG pathway analysis of target genes showed significant involvement of Hippo, FoxO, TGF-β, and p53 signaling pathways, which play a crucial role in RPL. Top 10 identified hub genes (*NFKB1*, *IL6*, *JUN*, *FOS*, *CXCL8*, *PTGS2*, *TGFB1*, *MMP9*, *STAT1*, and *CD4*) were significantly enriched in immunological pathways—Th1/Th2/Th17 differentiation, NF-κB pathway, TNF-α signaling, IL-17 signaling pathway, and vascular endothelial growth factor (*VEGF*) pathway.

**Conclusion:**

These results suggest that circulating EV-miRNAs in maternal blood could provide clinical insights into the pathogenesis of RPL and dysregulated immunological and molecular pathways at feto-maternal interface.

## 1 Introduction

Recurrent pregnancy loss (RPL) is defined as two or more consecutive losses of clinical pregnancy before 22 weeks of gestational age, affecting approximately 1%–2% couples worldwide (1). RPL is a heterogenous disease with multiple risk factors, which could be hormonal and metabolic disorders, uterine anomalies, cytogenetic abnormalities, and various immunological and genetic factors. In 50% of the cases, cause is unknown; hence, the exact etiology of RPL pathogenesis is yet to be elucidated (2).

Molecules involved in embryo–maternal crosstalk are not limited to certain growth factors, hormones, or cytokines. The discovery of microRNAs (miRNAs), small non-coding RNAs that regulate gene expression, has revolutionized our understanding of many biological processes, including pregnancy (3). Recently, the role of miRNAs has been implicated in the pathophysiology of RPL (4, 5). During pregnancy, these miRNAs play an important role in placental development through trophoblast cell proliferation, migration, and invasion, and in angiogenesis during placentation (6). These miRNAs expressed in trophoblast cells are secreted into the maternal circulation bound within extracellular vesicles (EVs) or exosomes, where they function in placental–maternal signaling (7). EVs are small vesicles, sized 30–150 nm in diameter, containing multifaceted cargoes and play an important role in regulation of various biological, molecular, and physiological processes (8). The role of these EV-miRNAs is well established in cancer diagnosis and therapeutics (9); however, their role in pregnancy, early detection, and monitoring of pre-eclampsia (PE) and RPL is still being explored (10–12). These EV-miRNAs released at the feto-maternal interface acts as nexus between immune modulation, trophoblast cell proliferation, invasion, and differentiation into decidual cells, and apoptosis. These miRNAs reach maternal circulation and regulate various genes, their molecular pathways, and cytokines, essential to sustain a successful pregnancy (13–16). Furthermore, in the case of idiopathic RPL, dysregulated immune response has been proposed as an important factor during pre-implantation, implantation, and maintenance of pregnancy (17).

Despite extensive research in the past decade, the exact pathogenesis of RPL is still not known, especially in idiopathic cases. Lack of reliable molecular marker and heterogeneity in RPL makes it even difficult to diagnose early in clinical settings. With the successful establishment of role of EV-miRNAs in fetal–maternal communication during normal and complicated pregnancy, there is a need to explore the potential of these EV-miRNAs as non-invasive biomarkers in RPL. The present study was conducted with an aim to amalgamate the existing evidence of EV miRNAs in pregnancy and its related disorders, with the differentially expressed (DE) miRNAs in pregnant women with idiopathic RPL and gestational-age-matched pregnant controls. This study emphasizes on the role of EV-miRNAs as non-invasive biomarkers for early diagnosis, prognosis, and treatment of RPL. Furthermore, it provides insights into various genes and related pathways, which had clinical implications and could be key players for RPL pathophysiology.

## 2 Materials and methods

### 2.1 Study population

**Figure 1:**
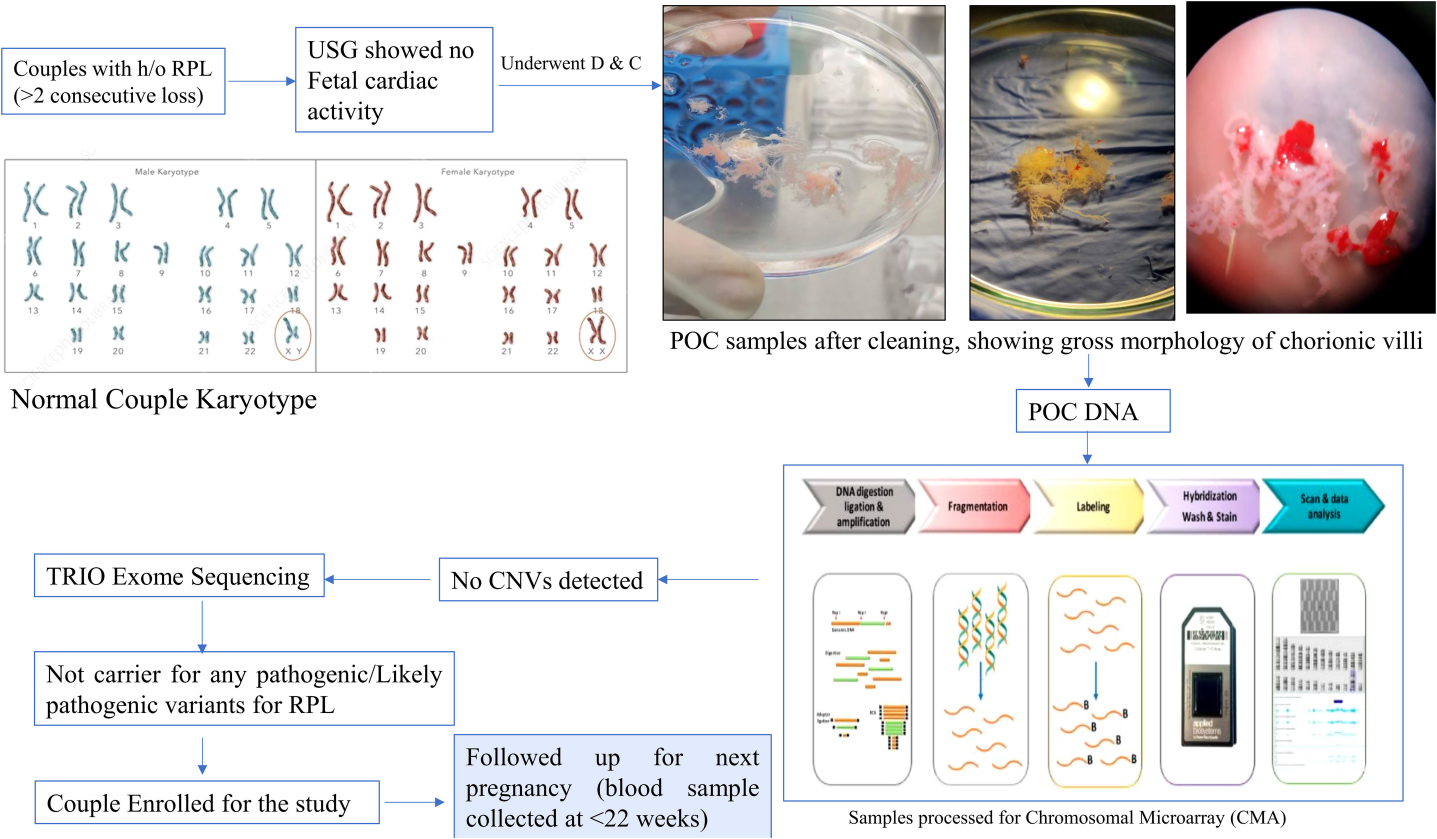
Flow chart showing complete genetic workup of study participants.

In present study, we included 10 pregnant women with history of RPL (≥2 abortions and no live births) with no known cause of RPL identified (no known uterine and endocrine anomalies or infections and no genetic alterations). Genetic analysis was done in each patient’s product of conception (POC) sample using chromosomal microarray (CMA), and patients negative for CMA underwent TRIO Exome sequencing (18). Patients with no causative variant were then followed up till their next pregnancy, and the blood sample was collected at ≤22 weeks of gestational age. The control group included five gestational-age-matched women with healthy pregnancy and at least one alive healthy child. The study was approved by the Institutional Ethics Committee. An informed consent was obtained from each participant before enrolling for the study. Baseline characteristics, such as age, clinical parameters (B.P., T3/T4/TSH, and APLA), number of pregnancies, gestational age, number of deliveries, and abortion history were collected from each patient. The flow chart is given as **Figure 1**.

### 2.2 Biological sample collection

Peripheral blood samples (~5–6 ml) were collected from each patient in an EDTA vial on ice and processed within 30 min for serum separation. The samples were centrifuged at 4,000×*g* for 15 min at 4°C, and serum was stored at −80°C till further processing.

### 2.3 EV isolation

EVs were isolated from 2 ml of serum using the Total Exosome Isolation Kit (for plasma) from Thermo Fisher Scientific, USA as instructed by the manufacturer. Briefly, serum samples were centrifuged at 2,000×*g* for 20 min to remove residual cells and debris. Next, one-half volumes of PBS and one-third volumes of exosome isolation reagent were added to the supernatant, and specimens were incubated at 4°C for 30 min before precipitation by centrifugation at 10,000×*g* for 5 min. Then, the exosome precipitate was reconstituted in PBS and stored at −20°C until further use.

### 2.4 EV characterization

#### 2.4.1 Transmission electron microscopy

A high-resolution transmission electron microscope (Jeol, JEM-2100Plus, Japan) was used to study the ultrastructure of exosomes. Briefly, exosomes were diluted with PBS and then loaded on a carbon formvar grid and negatively stained with 2% phosphotungstic acid. The exosomes were then visualized at 80 kV.

#### 2.4.2 Nanoparticle tracking analysis

The particle size distribution and concentration were detected using NTA. Exosomes were diluted with PBS (1:1,000) and then injected into the instrument capillary. NTA measurements were performed using a NanoSight NS300 instrument (NanoSight NTA 3.3; Malvern, Worcestershire, UK). The particle movement was recorded at camera level of 6 and camera shutter speed 86 ms and kept consistent between samples. The recorded videos were then analyzed for particle size (mean ± SD) and concentration (particles/ml).

#### 2.4.3 Western blotting for cell surface markers

The isolated EVs were pelleted and protein extracted using RIPA lysis buffer, and protein concentration was estimated using BCA Protein Assay Kit (Thermo Fisher Scientific). 20 ug of EV-protein was loaded on precast 4-20% polyacrylamide Mini-PROTEAN TGX gels (Bio-Rad Laboratories, CA, USA) and then transferred on a nitrocellulose membrane. The membrane was blocked in 5% BSA and then incubated in primary antibodies: CD63 (Thermo Fisher, #10628D), Flotillin-1 (Invitrogen, #PA5-17127), and PLAP (Invitrogen, #MA1-19354) (overnight). The blot was then incubated in secondary antibody, HRP-goat-anti-mouse (BD Biosciences) and acquired using Enhanced Chemiluminescence (ECL) Kit, (Bio-Rad, Hercules, California, USA) at Azure Biosciences Gel Doc System.

### 2.5 EV RNA isolation

The total RNA was extracted from the EVs using Total Exosome RNA and Protein Isolation Kit (Cat. 4478545, Invitrogen, CA, USA) using the manufacturer’s protocol. The RNA was quantified using Tape Station System (Agilent Technologies, Santa Clara, USA).

### 2.6 miRNA library preparation and sequencing

These samples were then processed for sequencing. The miRNA sequencing library was constructed using the QIAseq miRNA Library Kit (Qiagen, Germany). Briefly, to prepare the miRNA library, total RNA of each sample was used. 3′-and 5′-adaptor ligations were followed by cDNA synthesis, PCR amplification, and gel purification. Quantification was done with Qubit (Thermo Fisher, USA), and quality assessment was done using Agilent D1000 ScreenTape System in a 4150 TapeStation System. The libraries were then sequenced using Illumina NovaSeq 6000 platform (Illumina, USA).

### 2.7 Identification of differentially expressed miRNAs

The adapter sequences, low-quality bases, and reads shorter than 17 bp were removed using Trim-galore v0.6.6 and fastp program. The QC passed reads were mapped onto indexed human reference genome (GRCh38.p13) using mapper.pl script of miRDeep2 v2.0.1.2. Reads mapped to the reference genome were used to identify miRNAs with miRDeep2 using known and novel miRNA identification parameters. Human mature miRNAs from miRbase v22.1 were used for miRNA prediction. miRNAs with miRDeep score< 1 were excluded from the analysis resulting in total 1,569 novel and 679 known miRNAs. Expression levels of miRNAs were estimated using miRDeep2 quatifiler.pl script. Differential expression analysis was carried out using DESeq2 package after normalizing the data using relative log expression normalization method. miRNAs with absolute log2 fold change ≥ 1 and p-value ≤ 0.05 were considered significant.

### 2.8 Target prediction and pathway analysis

TargetScan (http://www.targetscan.org/vert_72/), miRanda (http://microRNA.org), miRDB (http://www.mirdb.org/cgibin/search.cgi), and starBase (http://starbase.sysu.edu.cn/) were used to predict target genes.

The biological processes that are involved with the DEGs, along with the functional enrichment analysis, were studied using the BINGO plug-in of Cytoscape. A hypergeometric test was carried out using Benjamini and Hochberg FDR correction. The Gene Ontology (GO) biological process was selected as the ontology file for executing enrichment analyses.

### 2.9 Protein–protein interaction network analysis and hub genes identification

Protein–protein interaction (PPI) network analysis was performed using the Search Tool for the Retrieval of Interacting Genes (STRING) database (https://string-db.org/). To assess possible PPI correlations, previously identified DEGs were mapped to the STRING database, followed by the extraction of PPI pairs. Cytoscape software v3.9.0 (https://cytoscape.org/) with the CytoHubba plugin was then employed to visualize the PPI network and to identify hub genes based on the maximal clique centrality (MCC). MCC is a sensitive algorithm for detecting essential genes, as it measures how many maximal cliques a node belongs to—reflecting both connectivity and influence within a network. These hub genes were then subjected to CytoScape plugins ClueGo and CluePedia for pathway analysis.

### 2.10 Statistical analysis

Continuous variables are presented as mean ± standard deviation (SD) for normally distributed data or as median and interquartile range. We compared the normally distributed data using the Student’s t-test, and Mann–Whitney U-test was used for non-normally distributed data. A p-value < 0.05 was considered to be statistically significant. All analyses were performed using SPSS software (version 29, Chicago, IL, USA).

### 2.11 Validation of significant exosomal-miRNA from literature

To increase the generalization and external validity of the results obtained here, we further validated the intersection of various genes controlled by differently expressed EV-miRNAs using the recently published studies (11, 12, 19). We used the search term “recurrent pregnancy loss/recurrent spontaneous abortions/implantation failure” and “exosomal/EV miRNAs” from PubMed. We included the datasets wherein the gene expression profiling was done using miRNA sequencing, and the maternal blood sample was collected within first trimester. FunRich tool was used for target enrichment and Venn diagram preparation.

## 3 Results

### 3.1 Clinical characteristics of patients

There were 10 pregnant women (≤22 weeks of gestation) with history of RPL and five subjects with healthy pregnancy and at least one live healthy birth as controls. The clinical characteristics of these patients and controls are listed in **Table 1**. No statistically significant correlation was observed between age, number of abortions, gestational age, and number of pregnancies and deliveries in women with RPL and controls (**Table 1**).

**Table 1:** Demographic and clinical characteristics of patients and controls.

Characteristics	RPL (n = 10)	Control (n = 5)	p-value
Age (years)	31.60 ± 2.63	28.80 ± 1.92	0.056
Gestational Age (weeks)	8.07 ± 3.15	6.74 ± 1.76	0.401
Number of Pregnancies	5.20 ± 2.10	2.20 ± 0.45	0.008
Number of Abortions/Losses	4.00 ± 1.63	0.00 ± 0.00	<0.001
Number of Deliveries	0.00 ± 0.00	1.20 ± 0.45	<0.001

### 3.2 EV Isolation and characterization

**Figure 2:**
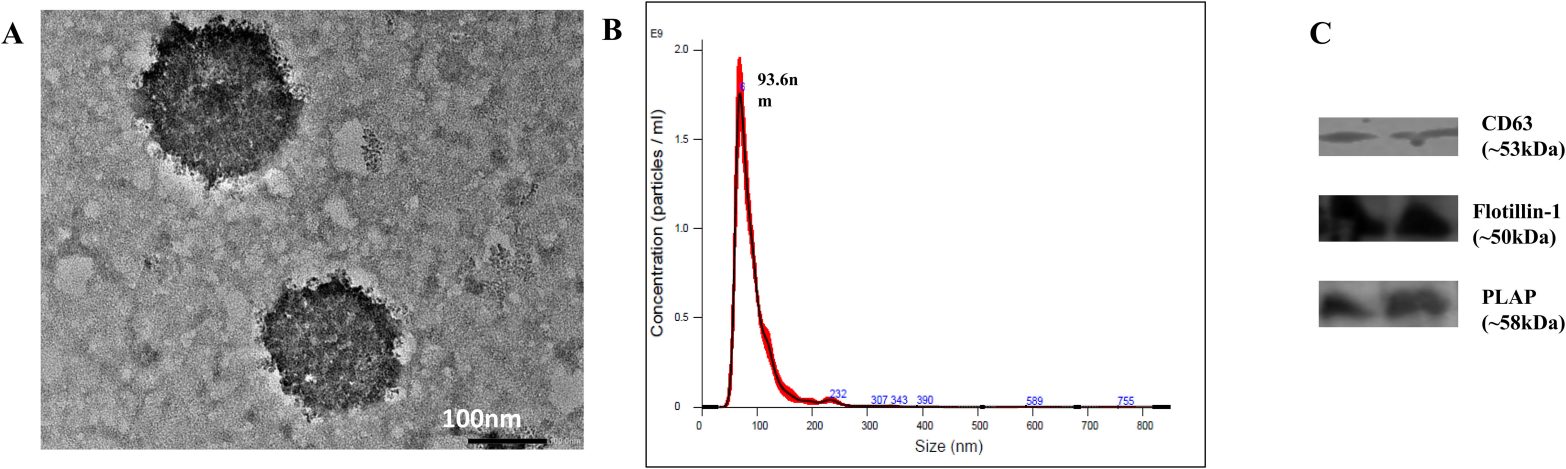
Characterization of EVs isolated from serum samples. **(A)** Morphology of EVs as viewed under HR-TEM. **(B)** NTA showing particle size and concentration of exosomes. The size of EVs ranged from 50 to 200 nm, with D90 of 132.4 nm and mean size of 94 ± 1.9 nm, and concentration of 7.48 × 10^10^ ± 4.05 × 10^9^ particles/ml. **(C)** Western blot showing the presence of cell surface marker CD63, flotillin-1 and Placental alkaline phosphatase (PLAP).

TEM, NTA, and WB analysis was done to characterize the isolated EVs. HR-TEM showed that the isolated EVs had oval/round morphology (**Figure 2A**). NTA analysis showed that the size of EVs ranged between 50 nm and 200 nm, with a mean diameter size of 93.6 nm (**Figure 2B**). Furthermore, the NanoSight analysis demonstrated that the concentration of EVs was 7.48 × 10^10^ particles/ml. WB analysis confirmed the presence of exosomal cell surface marker CD63, flotillin-1, and placental alkaline phosphatase (PLAP) (**Figure 2C**).

### 3.3 Differential expression of miRNAs in patients and controls

**Figure 3:**
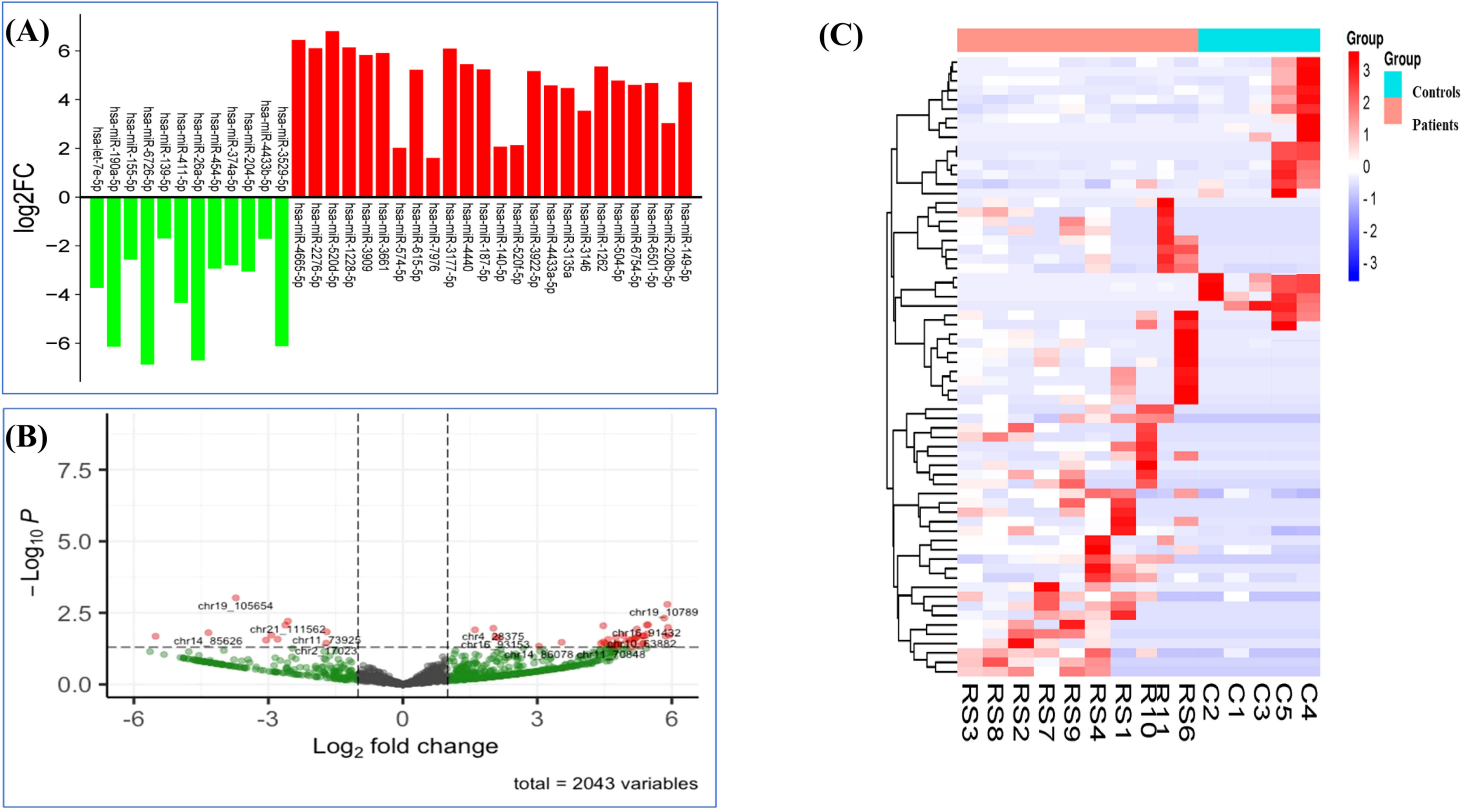
Differentially expressed miRNAs in RPL patients. **(A)** Expression profile of DE miRNAs in RPL patients and controls based on threshold of FC ≥ 1 and p < 0.05. **(B)** Volcano plot of DE miRNAs (chromosomal IDs). **(C)** Heatmap of 66 DE miRNAs in RPL patients and controls (p <0.05).

A total of 2,043 miRNAs were identified, among which 559 were known and 1,484 were novel miRNAs. Among these miRNAs, 66 were significantly differentially expressed (p <0.05), wherein 44 miRNAs were known and 22 were novel miRNAs. Their accession IDs and sequences are mentioned in **Supplementary Table S1**). Out of the significantly expressed miRNAs, 37 were downregulated and 29 were upregulated, log_2_|FC| ≥ 1 (**Table 2**, **Figures 3A, B**). The hierarchical clustering analysis of the 66 miRNAs is depicted with the heatmap. The color scale, ranging from red to blue, indicated high to low expression of each miRNA transcript. (**Figure 3C**).

**Table 2:** Statistically significant differentially expressed miRNAs between women with RPL and controls.

Downregulated miRNAs			
ID	mature miRBase miRNA	log2 Fold Change	p-value
chr19_105654	hsa-let-7e-5p	−3.7254	0.000951
chr15_88692	hsa-miR-190a-5p	−6.14015	0.002346
chr21_111562	hsa-miR-155-5p	−2.56317	0.006191
chr1_5388	hsa-miR-6726-5p	−6.87092	0.011997
chr11_73925	hsa-miR-139-5p	−1.69882	0.014632
chr14_85626	hsa-miR-411-5p	−4.33881	0.015623
chr3_21034	hsa-miR-26a-5p	−6.69882	0.015752
chr17_100259	hsa-miR-454-5p	−2.93218	0.018971
chrX_119079	hsa-miR-374a-5p	−2.79361	0.026589
chr9_62118	hsa-miR-204-5p	−3.0526	0.028441
chr2_17023	hsa-miR-4433b-5p	−1.70797	0.036151
chr15_91109	hsa-miR-3529-5p	−6.12229	0.03703
Upregulated miRNAs			
chr9_58746	hsa-miR-4665-5p	6.45042616	9.4927E-05
chr13_80875	hsa-miR-2276-5p	6.11003569	0.00020524
chr19_105778	hsa-miR-520d-5p	6.80646908	0.00353588
chr12_76390	hsa-miR-1228-5p	6.14175588	0.00388438
chr22_113414	hsa-miR-3909	5.83234459	0.00481849
chr5_36010	hsa-miR-3661	5.91361551	0.01016591
chr4_28375	hsa-miR-574-5p	2.01899306	0.01095652
chr12_76269	hsa-miR-615-5p	5.22167064	0.01168123
chr3_26163	hsa-miR-7976	1.60991598	0.01245491
chr16_91545	hsa-miR-3177-5p	6.0909665	0.01617725
chr2_20257	hsa-miR-4440	5.45184424	0.01725977
chr18_102896	hsa-miR-187-5p	5.23933664	0.02136137
chr16_93153	hsa-miR-140-5p	2.06807824	0.02230465
chr19_105740	hsa-miR-520f-5p	2.13507763	0.0236734
chr12_77263	hsa-miR-3922-5p	5.16763966	0.02617872
chr2_12254	hsa-miR-4433a-5p	4.58265841	0.02670685
chr3_20754	hsa-miR-3135a	4.47560728	0.03286033
chr7_50322	hsa-miR-3146	3.54261837	0.03376134
chr1_7330	hsa-miR-1262	5.35889385	0.03666333
chrX_119995	hsa-miR-504-5p	4.7792789	0.04093006
chr11_70922	hsa-miR-6754-5p	4.60704195	0.043421
chr21_111736	hsa-miR-6501-5p	4.68177473	0.04451979
chr14_86078	hsa-miR-208b-5p	3.03837182	0.04642803
chr2_15572	hsa-miR-149-5p	4.71261799	0.04867914

### 3.4 Functional annotation and pathway enrichment

#### 3.4.1 microRNA-target enrichment and network-based analysis

**Figure 4:**
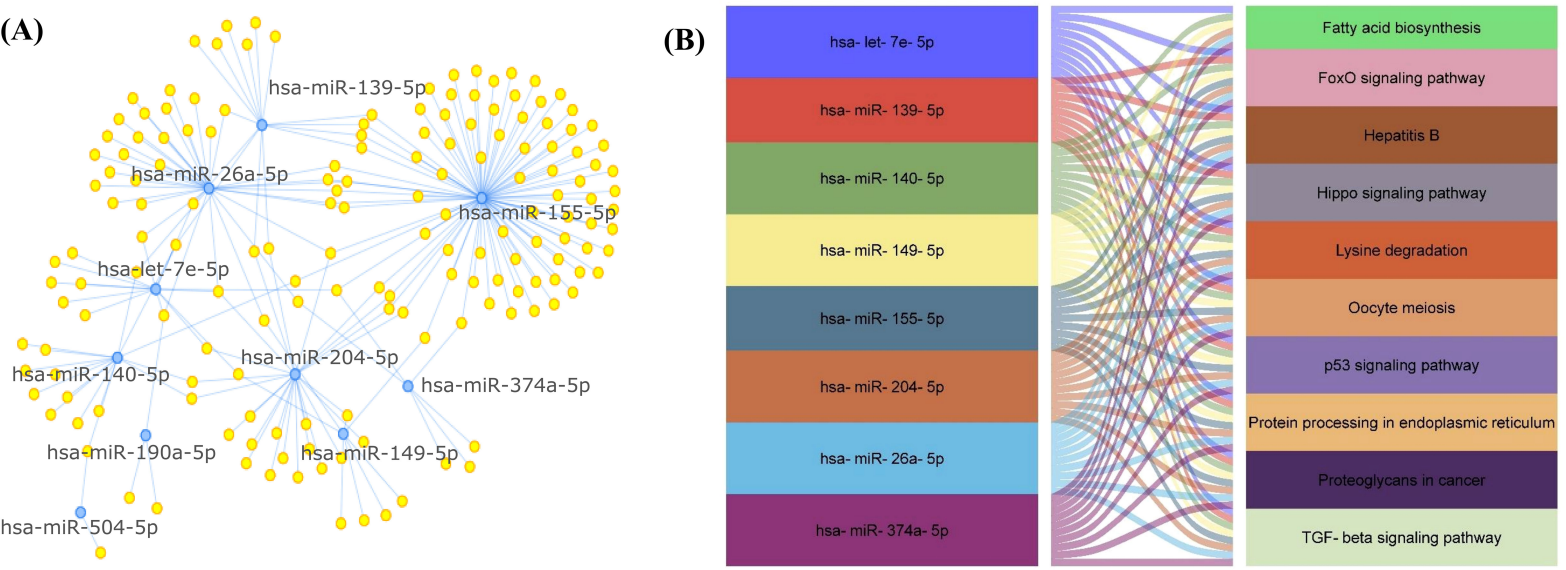
**(A)** Target enrichment showing miRNAs with highest degree of association/enrichment. **(B)** Snake plot showing KEGG pathway network for EV-miRNAs.

Significantly DE-miRNAs were subjected to target enrichment by miRtarBase for network-based analysis. The highest degree came for nine miRNAs (hsa-miR-155-5p, hsa-miR-26a-5p, hsa-miR-204-5p, hsa-miR-140-5p, hsa-miR-139-5p, hsa-let-7e-5p, hsa-miR-149-5p, hsa-miR-374a-5p, and hsa-miR-190a-5p) (**Table 3**, **Figure 4A**). Furthermore, these miRNAs were selected for pathway analysis.

**Table 3:** Target enrichment for differentially expressed miRNAs for network-based analysis.

Node	Degree	Closeness	Betweenness	Eccentricity	Clustering Coefficient	Average Shortest Path Length
hsa-miR-155-5p	87	0.496	0.668	5	0	2.01
hsa-miR-26a-5p	33	0.385	0.272	5	0	2.58
hsa-miR-204-5p	25	0.361	0.188	5	0	2.75
hsa-miR-140-5p	15	0.343	0.134	5	0	2.9
hsa-miR-139-5p	15	0.34	0.0903	5	0	2.92
hsa-let-7e-5p	14	0.356	0.0996	5	0	2.8
hsa-miR-149-5p	8	0.314	0.0501	7	0	3.17
hsa-miR-374a-5p	6	0.291	0.0344	7	0	3.42
hsa-miR-190a-5p	3	0.228	0.0217	7	0	4.37

#### 3.4.2 KEGG pathway

The KEGG pathway analysis showed that the DE-miRNAs in women with RPL were enriched in TGF-beta signaling pathway, Hippo signaling pathway, lysine degradation, hepatitis B, proteoglycans in cancer, fatty acid biosynthesis, oocyte meiosis, protein processing in endoplasmic reticulum, p53 signaling pathway, and FoxO signaling pathway (**Table 4**, **Figure 4B**).

**Table 4:** KEGG pathway analysis showing pathways associated with significant DE miRNAs.

KEGG pathway	p-value	Genes	miRNAs
TGF-beta signaling pathway	1.42E−09	44	8
Hippo signaling pathway	1.42E−09	71	8
Lysine degradation	3.87E−08	25	8
Hepatitis B	6.30E−08	70	8
Proteoglycans in cancer	2.57E−07	94	8
Fatty acid biosynthesis	4.59E−07	6	6
Oocyte meiosis	4.59E−07	56	8
Protein processing in endoplasmic reticulum	4.92E−07	85	8
p53 signaling pathway	1.60E−06	43	8
FoxO signaling pathway	2.62E−06	70	8

#### 3.4.3 Reactome and wiki pathways

**Figure 5:**
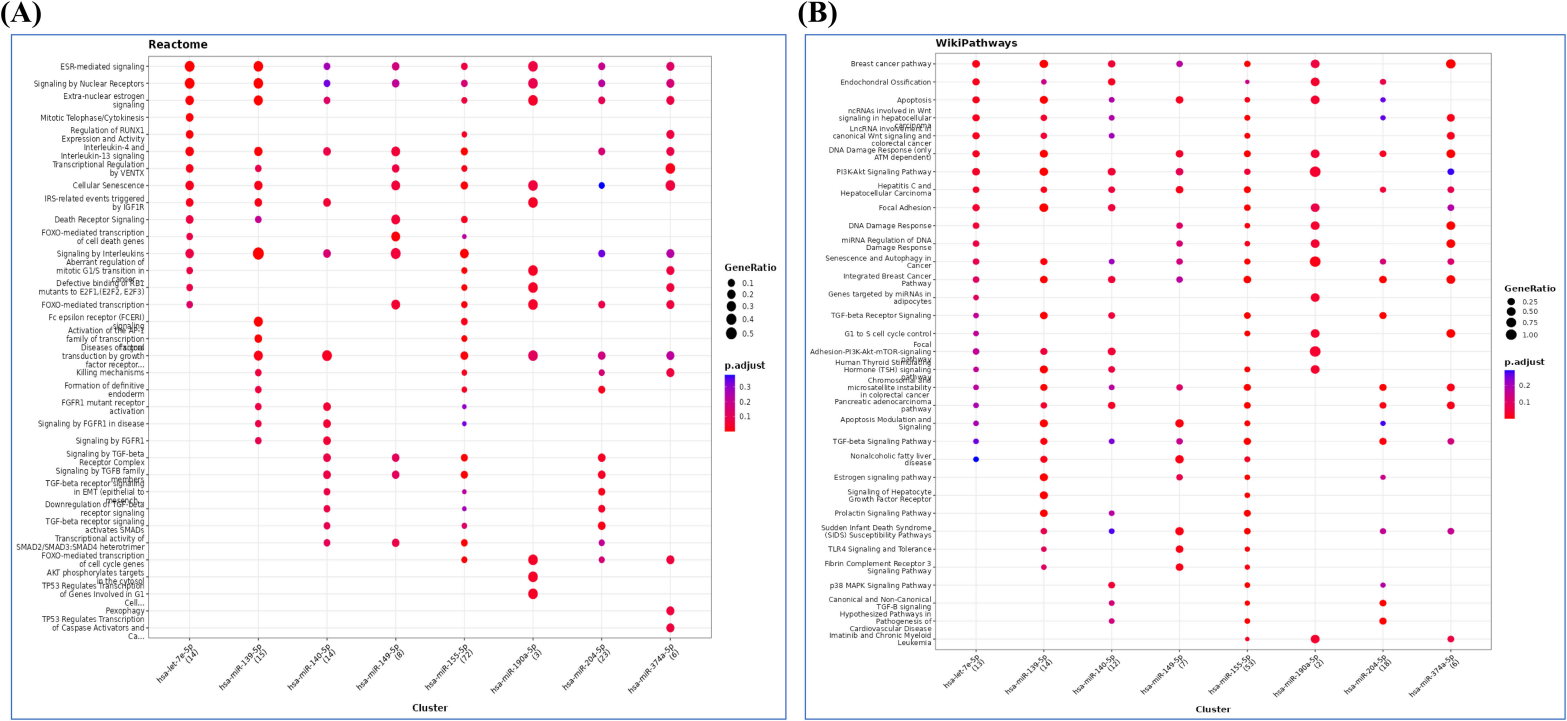
**(A)** Reactome and **(B)** wiki pathway analysis showing possible pathways related to pathophysiology of RPL.

The reactome pathway analysis further revealed the role of FoxO-mediated transcription, Akt phosphorylation, TP53 regulation of transcription, IL-4 and IL-13 signaling, and cellular senescence (**Figure 5A**). The major pathways depicted in wiki pathways, which correlated with RPL included PI3-Akt-mTor signaling pathway, TLR4 signaling and tolerance, TGF-β, and p38 MAPK signaling pathway (**Figure 5B**).

#### 3.4.4 miRNA target genes

These 66 DE-miRNAs were subjected to FunRich tool for target gene prediction, which resulted in 1,586 genes.

#### 3.4.5 GO and KEGG pathway analysis for the target genes

**Figure 6:**
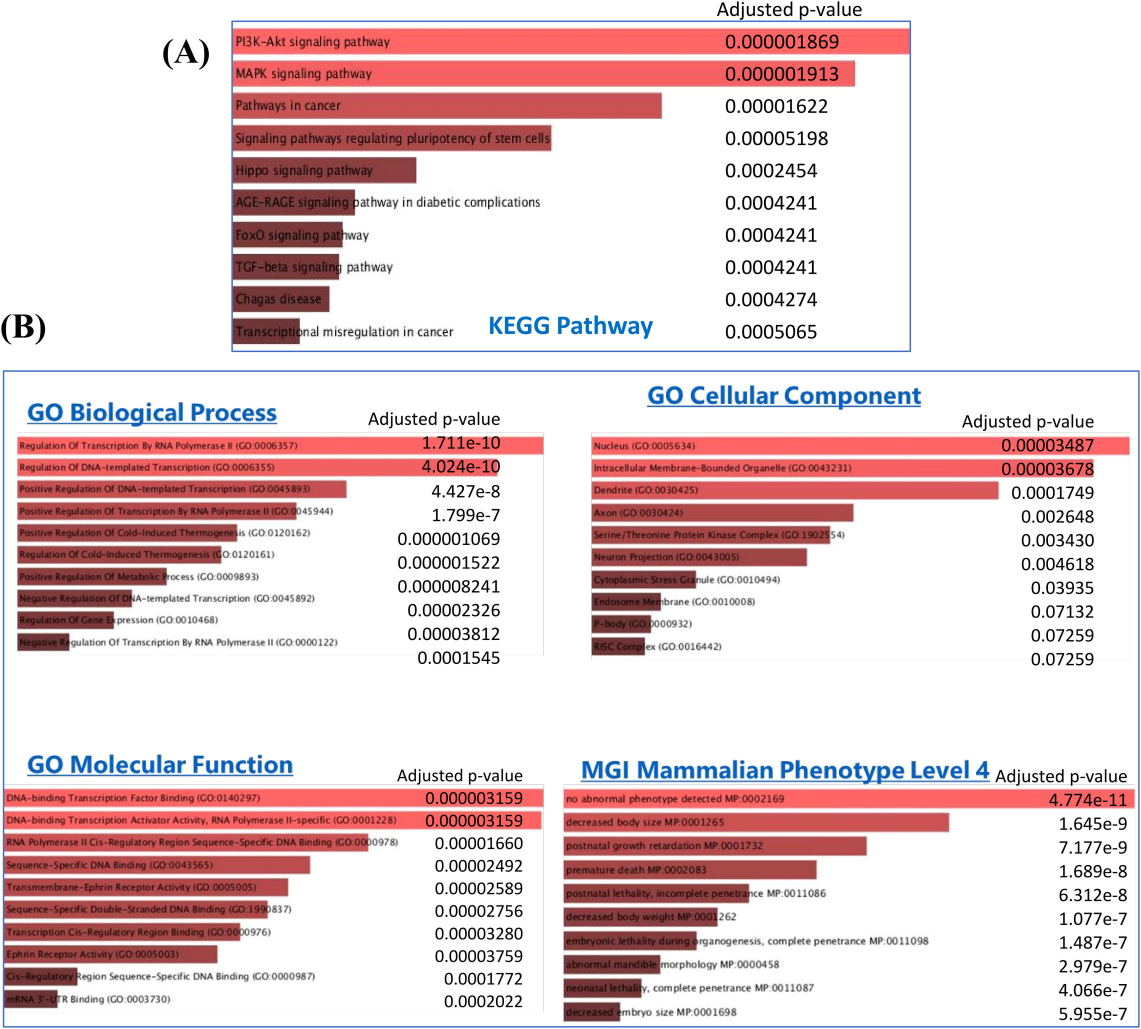
**(A)** KEGG pathway and **(B)** Gene Ontology (GO) analysis for 1,586 target genes obtained from the significant DE miRNAs.

Furthermore, 1,586 genes were included in GO and KEGG pathway analysis using Enrich tool (**Supplementary Table S2**, **Figure 6**). The GO consists of three subtypes: biological processes (BP), cellular processes, and molecular function. As shown in **Figure 6B**, biological-process-related results showed that these genes were mainly involved in the regulation of transcription, while the major cellular component included the nucleus, intracellular membrane-bound organelles, axon, and dendrites, and the molecular functions were also related to transcription factors and transmembrane activity. The KEGG pathway analysis showed that these genes were significantly involved in PI3K-Akt, MAPK, FoxO, and TGF-β signaling pathways, which plays a crucial role in RPL (**Supplementary Table S2**, **Figure 6A**).

The MGI Mammalian Phenotype showed significant correlation with post-natal growth retardation, premature death, embryonic lethality, neonatal lethality, and decreased embryo size These factors could alter the normal course of pregnancy and result in abortion.

#### 3.4.6 PPI network analysis and hub genes

**Figure 7:**
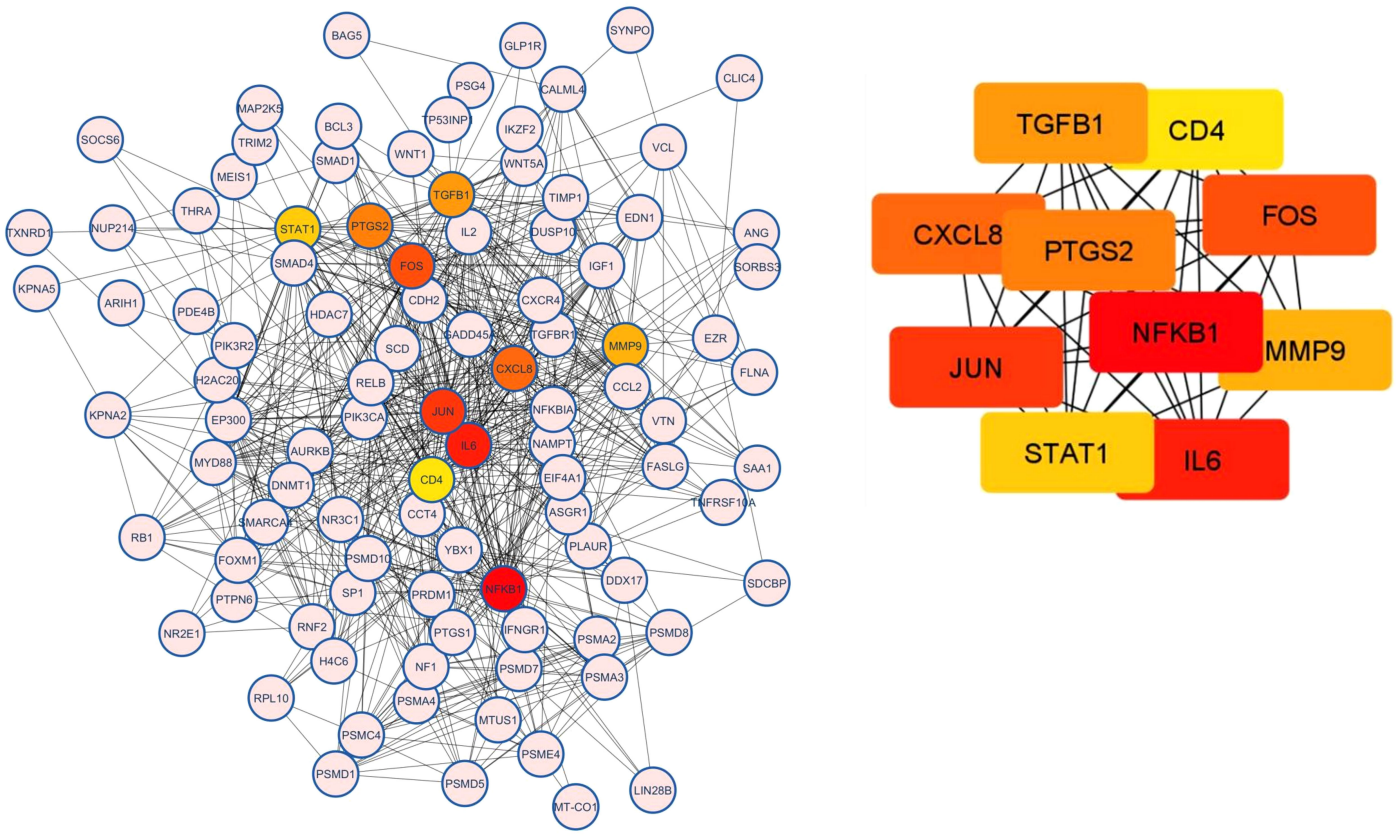
Protein–protein interaction (PPI) analysis and top 10 hub genes related to RPL based on the maximal clique centrality (MCC). Size and color representation of the gene nodes are arranged in decreasing order of MCC scores; the darkest and biggest node represent the high MCC score.

**Figure 8:**
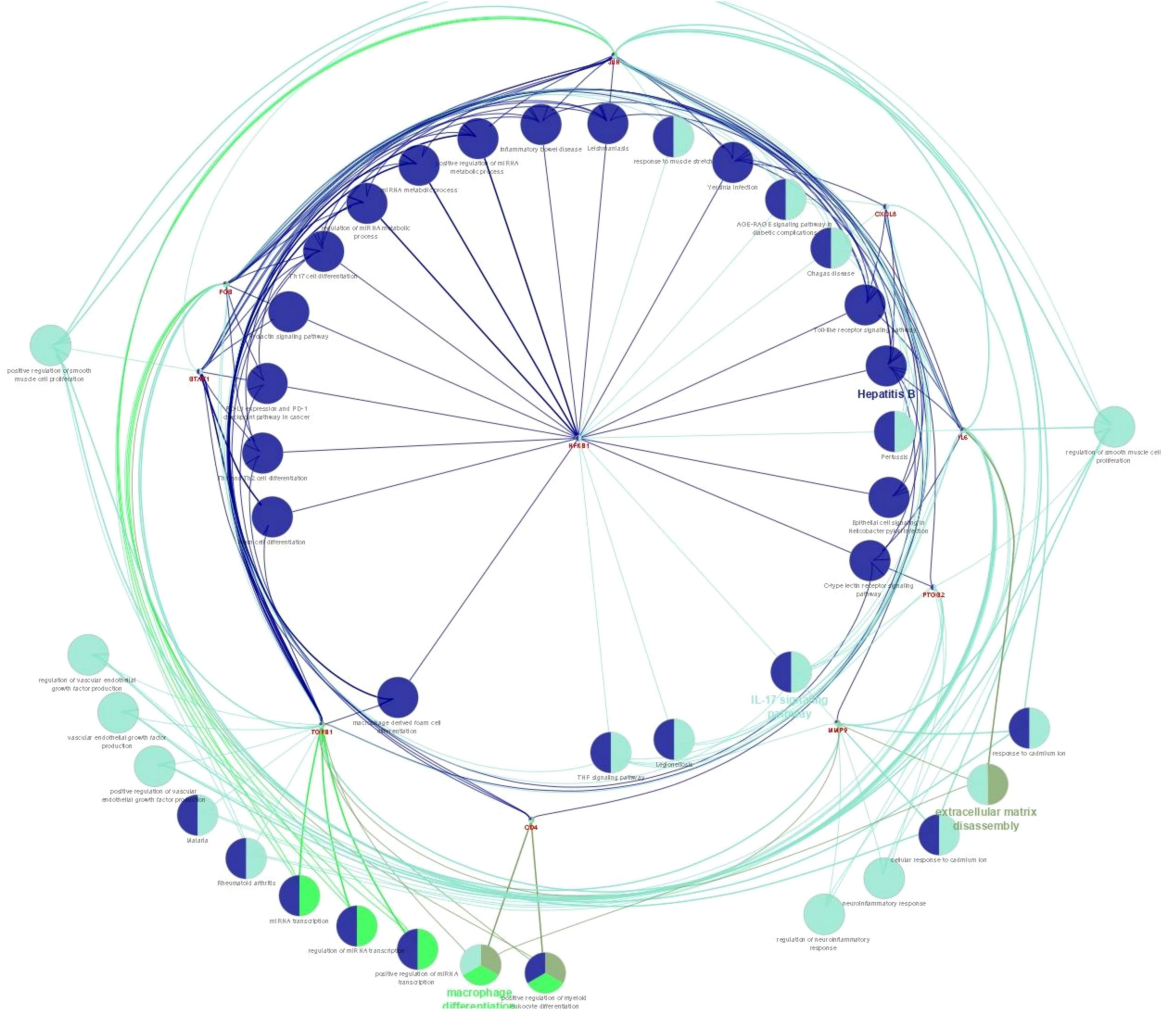
Pathway enrichment analysis for biological processes of hub genes of RPL-linked miRNAs derived using ClueGO (version 2.5.1) and Cluepedia (version 1.5.7) plugin in Cytoscape (version 3.8.2) environment.

PPI network analysis was performed, wherein previously identified DEGs were mapped to the STRING database, followed by the extraction of PPI pairs using Cytoscape cytoHubba plugin (**Figure 7**) based on the maximal clique centrality (MCC). Size and color representation of the gene nodes are arranged in decreasing order of MCC scores; the darkest and biggest node represent the high MCC score. In present study, we observed top 10 hub genes, namely, *NFKB1*, *IL6*, *JUN*, *FOS*, *CXCL8*, *PTGS2*, *TGFB1*, *MMP9*, *STAT1*, and *CD4* (**Supplementary Table S3**, **Figure 7**). We observed that these genes were associated with placentation, embryo implantation, development, and responsible for innate and adaptive immune response during pregnancy. Pathway analysis revealed that these genes are enriched with significant immunological pathways—Th1/Th2/Th17 differentiation, TNF-α signaling, IL-17 signaling pathway, and regulation of vascular endothelial growth factor (VEGF) (**Figure 8**, **Supplementary Excel Table S1**).

### 3.5 Validation of miRNAs from literature

**Figure 9:**
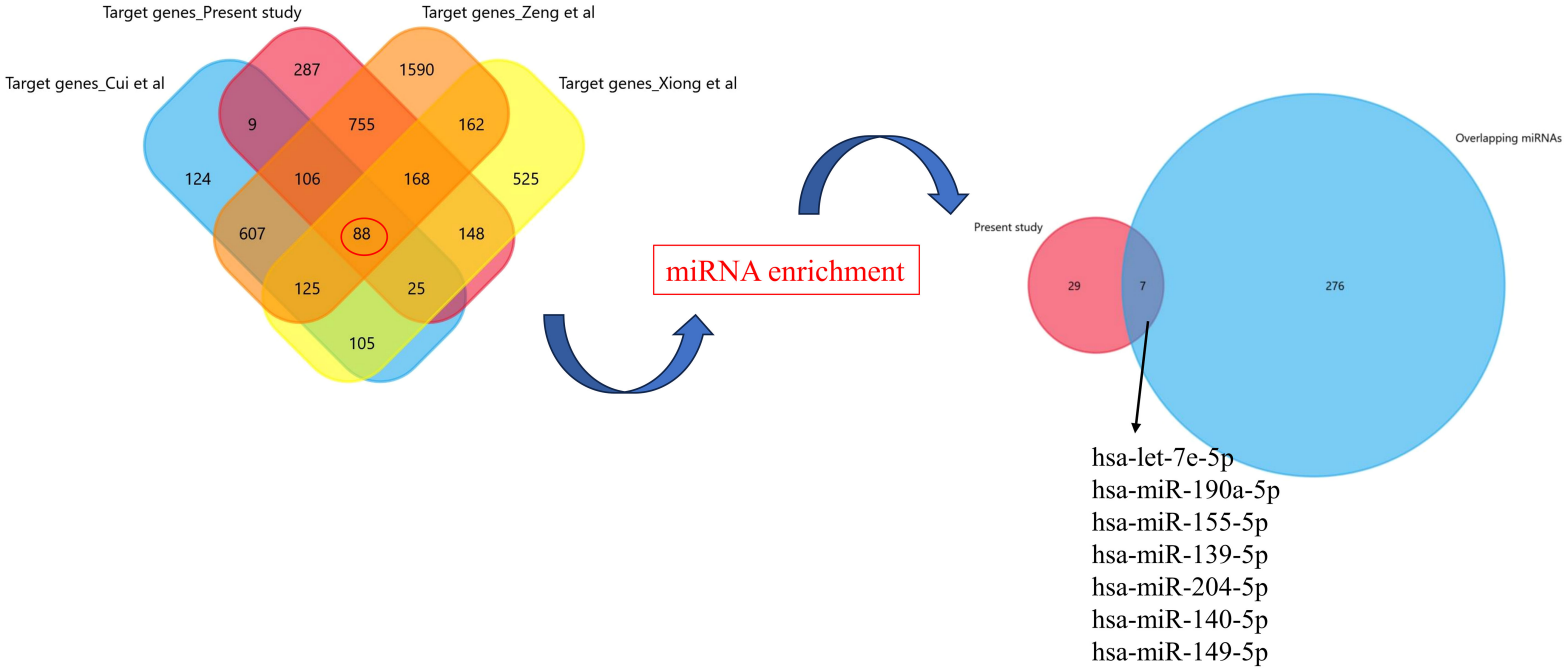
Enrichment analysis of target genes from the present study and available datasets.

After putting the keywords in PubMed, we ended up with three studies (11, 12, 19). We extracted the list of differentially expressed miRNAs from these studies and did the gene enrichment using FunRich tool. To see the common genes, we have compared the gene list of the present study with the previously published literature and found 88 common genes (**Figure 9**). Further target miRNAs of these genes were extracted and compared with 66 miRNAs of the present study, which revealed seven common miRNAs: hsa-let-7e-5p, hsa-miR-190a-5p, hsa-miR-155-5p, hsa-miR-139-5p, hsa-miR-204-5p, hsa-miR-140-5p, and hsa-miR-149-5p. The expression of these miRNAs was evaluated in our dataset. According to the expression profile, hsa-let-7e-5p, hsa-miR-190a-5p, hsa-miR-155-5p, hsa-miR-139-5p, and hsa-miR-204-5p showed significantly lower expression (p<0.05), while hsa-miR-140-5p and hsa-miR-149-5p showed significantly higher expression in the exosomes of the RPL group compared with the control group (p < 0.05) (**Supplementary Figure S1**).

### 3.6 Clinical correlation of selected miRNAs with patient characteristics of RPL

To determine the clinical correlation of miRNAs expression, we analyzed each selected miRNAs with RPL women clinical data. We tested the correlation of miRNAs expression with clinical factors, i.e., maternal age, gestational age, and number of pregnancy losses. For correlation with maternal age, we subdivided the age into two categories: <30 and >30 years. None of the miRNAs showed statistically significant differences between the two age groups. Gestational age was subdivided into <8 and >8 weeks; only miR-139-5p showed a statistically significant difference between the two gestational groups (p=0.024). Number of pregnancy losses was divided into three categories: ¾3 loss, 3–6 losses, and >6 losses. None of the miRNAs showed significant correlation with number of losses (**Supplementary Table S4**).

## 4 Discussion

At the time of pregnancy human placenta releases miRNA enclosed in EVs, which play an important role in migration, invasion, differentiation, and proliferation of trophoblast cells. Aberrant expression of these miRNAs could result in altered trophoblast function and angiogenesis, which in turn could lead to adverse pregnancy complications, such as RPL (10–12). There are very few studies wherein the role of EV-miRNAs is studied in RPL patients (11, 12); however, the role of circulating miRNAs in maternal blood of RPL patients has been widely studied (20–23), with few studies in animal model as well (24, 25).

In the present study, we found a total of 2,043 DE-miRNAs between patients with RPL and controls, out of which 66 were significantly differentially expressed. After doing the validation from other studies (11, 12, 19) on exosomal miRNAs in RPL, we ended up in seven intersecting miRNAs, which were differentially expressed (**Figure 9**). Interestingly, these were the same miRNAs that we found after target enrichment and network-based analysis of our significantly DE-miRNAs list (**Table 3**).

Exosomal-hsa-let-7e-5p was significantly downregulated in the present study. Kong et al. have shown that let-7e plays a vital role in the regulation of endothelial function and inflammation and regulates progenitor endothelial cell migration and tube formation (26). Later, Lin et al. found that let-7e regulates vascular endothelial cell function and inflammation via activation of the NF-κB pathway (27). Overexpression of hsa-let-7e leads to activation of Th1 and Th17 cells, which is linked with aggravation of coronary heart disease (28). Hosseini et al. observed that hsa-let-7e was downregulated in the maternal plasma of early pregnancy loss patients. hsa-let-7e increases the expression of anti-apoptotic protein Bcl-x1, which disrupts the apoptotic mechanism during placentation and embryonic development; this in turn induces miscarriages (29). Furthermore, there have been studies that reported differential expression of other variants of let-7, such as let-7c (29, 30) and let-7a, let-7d (21) in RPL patients.

Another important miRNA was miR-155, which has been shown to regulate apoptosis via targeting caspase 3 and NF-κB signaling (31). In a study by Li et al. and Zhang et al., it was observed that miR-155 was significantly upregulated in the decidua of RPL patients (32, 33), while no significant change was observed in the villous tissue of RPL patients by Geng et al. (34). miR-155 may play an important role in the development of PE through the downregulation of CYR61; Firatligil et al. showed a significant decrease in CYR61 gene expression in RPL (35). In the present study, we observed that miR-155-5p was significantly downregulated, which is in line with the study by Zhang et al.,wherein they observed that miR-155-5p was downregulated in both serum and decidua of RPL patients and plays an important role in growth and proliferation of decidual stromal cells and inhibit their apoptosis by inhibiting the NF-κB signaling pathway (36). Yan et al. also observed that miR-155 levels were significantly downregulated in T-cell subset of RPL patients (37). Downregulated levels of miR-155 in the blood of RPL patients have been widely reported (38, 39).

In a recent *in vivo* study by Liu et al., the role of miR-139-5p was studied in pre-eclampsia mice model. They observed that mesenchymal stem-cell-derived exosomal-miR-139-5p plays a crucial role in trophoblast cell migration, invasion, and proliferation, via activating the ERK/MMP2 pathway and downregulating PTEN (14). Another study has shown that in severe pre-eclampsia (PE) patients, miR-139-5p was downregulated and negatively correlated with soluble fms-like tyrsine kinase-1 (sFlt-1) expression (40). Our study is the first one to report decreased expression of exosomal-miR-139-5p in RPL patients. Since the role of miR-139-5p is well established in PE, we speculate that the molecular mechanism of miR-139 is similar in RPL, i.e., regulation of proliferation and migration of trophoblast cells. miR-26a-5p plays a crucial role in early pregnancy by synchronizing the function of genes and processes regulating the implantation and development of the embryo and trophoblast cell function (41). In the present study, we observed that exosomal-miR-26a-5p was significantly downregulated in RPL patients. The exact role of miR-26 in RPL is still not established; however, it has been studied in the case of PE patients. miR-26a has been reported to be significantly upregulated in the pre-eclamptic patient’s plasma (42).

During pregnancy miR-374a-5p is overexpressed, and its level increases with advancing gestational age (43). Previous studies have shown that miR-374a-5p has been associated with PE (44), pre-term birth (45), and intra-uterine growth restriction (46). In their study, Cui et al. observed that levels of miR-374a-5p were significantly downregulated in plasma-derived EVs from maternal circulation of spontaneous abortion patients. The results of our study corroborated with the study by Cui et al. (12). Although the exact role of miR-374 during pregnancy is not clear, it could be interpretated that as it is associated with downregulation of pro-inflammatory markers, which might lead to pregnancy-associated complications. Furthermore, in this study, the expression of exosomal-miR-204-5p was significantly downregulated in maternal circulation. Our results are in line with the studies by Cui et al. (12) and Qin et al. (5), while Yu et al. showed that overexpression of miR-204 leads to inhibition of trophoblast cell proliferation and inhibition by targeting MMP-9 (47).

In the present study, we observed that exosomal-miR-149-5p was significantly upregulated in RPL patients than the control. miR-149 has been demonstrated to regulated genes involve in endothelial dysfunction (48). Furthermore, its overexpression promotes cell apoptosis and inhibits the activity of trophoblasts (cell invasion and migration). In an *in vitro* study, Wang et al. demonstrated that knockdown of miR-149 halted the development of recurrent miscarriage via upregulating the expression of *RUNX2* gene in chorionic tissues of pregnant women and activating the PTEN/Akt signaling pathway (17). A study by Whigham et al. showed that combined expression of miR-149 with miR-363 in maternal circulation, at 36 weeks of gestational age, could be used as biomarker to detect pre-eclampsia with a sensitivity of 45% (49). A recent meta-analysis by our group has also shown a positive association between miR-149 polymorphism and increased susceptibility with RPL (50). Zeng et al. has also shown that miR-149-5p is overexpressed in patients with recurrent implantation failure (19). Furthermore, in the present study, we observed a significant association between pregnancy loss and exosomal miR-190a (downregulated) and exosomal miR-504 (upregulated). However, no literature was found for miR-190a-5p and miR-504-5p related to their role in pregnancy or pregnancy-related disorders.

During pregnancy, miRNAs from chromosome 19 and chromosome 14 cluster (C19MC and C14MC) are released from the trophoblast cells, wherein they are known to play an important role in cell proliferation, invasion, and differentiation, and circulate in maternal blood (7). We have also observed significant miRNAs from C19MC (let-7e-5p, miR-520d-5p, miR-411-5p, and miR-520f-5p) and C14MC (miR-208b-5p). Similar to our study miR-520 was upregulated in the blood/plasma (51), decidual tissue (52), and villus tissue (53) of RPL patients. Additionally, in the present study, most of the identified hub genes (*NFKB1*, *IL6*, *JUN*, *FOS*, *CXCL8*, *PTGS2*, *TGFB1*, *MMP9*, *STAT1*, and *CD4*) were related with IL-17 signaling and hepatitis B pathways, which are further associated with immune functioning through TH1/TH2 and TH17 cell differentiation, TNF signaling pathway, NFκB pathway, and regulation of VEGF, during pregnancy and their effect on the uterine microenvironment.

Nuclear factor kappa B (NF-κB) is an important transcription factor, which is involved in regulation of immunogenic response during pregnancy. Excessive activation of NF-κB leads to placental hemorrhage and increased trophoblast apoptosis, leading to disorders associated with the development of fetus, such as abortion, intrauterine growth restriction, or pre-term birth (54, 55). Furthermore, NF-κB regulates the transcription of IL-6, which is a crucial cytokine for the successful maintenance of pregnancy (56). It blocks Treg cells formation and induce Th17 cells differentiation. Another important regulator of uterine and vascular remodeling during pregnancy is matrix metalloproteinases (MMPs). Especially MMP-2 and MMP-9 have been shown to play an important role in placentation, vasodilation, and uterine expansion (57). The findings from a recent study by Karachrysafi et al. showed that MMP-9 levels were increased in both the decidua and trophoblast tissue from RPL women. This increase in MMP-9 levels could be due to exaggerated immune response and trophoblastic invasion at feto-maternal interface as part of RPL pathogenesis (58). Furthermore, on doing pathway analysis of these miRNAs, we observed Forkhead box transcription factors (FoxO) signaling, Hippo signaling, transforming growth factor beta (TGF-β) signaling, and p53 signaling pathways as some of the critical pathways that have been regulated by the potential miRNA biomarkers of RPL. FoxO1 is critical for placental morphogenesis and governs cell-to-cell communication for chorioallantois attachment. Knockdown of FoxO1 in mice model disrupts the feto-maternal cross-talk, which in turn caused implantation failure and infertility in mice (59). Hippo signaling pathway is highly conserved and regulates organ size, tissue development and growth, cell fate, regeneration, stemness, and differentiation (60). The tumor suppressor protein p53 plays a crucial role in maintaining somatic cell genomic stability and prevention of tumor (61). It appears to play a similar role in human fertility. The p53 pathway monitors oocyte genomic quality and regulates reproduction at the implantation stage of the embryo (62). In early pregnancy, TGFβ is present in the extracellular matrix of villi and decidual tissue and plays an important role in the functional regulation and development of placenta (63). Dirisipam et al., in their study, observed that circulating levels of TGF-β is less in RPL patients as compared to controls (64). Furthermore, Lu et al. showed in an *in vitro* model that TGF-β increases vascular maturation in women with RPL, and thus, manipulation of TGFβ1 pathway maybe a viable treatment option for such patients (65). In addition, our study showed that EV-miRNAs at the maternal–fetal interface affects the immune functioning via controlling the expression of several genes and related pathways, which may provide more evidence supporting the exploration of the possible mechanisms of RPL.

## 5 Limitations

However, there are certain limitations that needs to be addressed. First of all, this study has a relatively smaller sample size; thus, the results need to be replicated in larger population. Efficient and consistent extraction of EV-miRNAs is a significant bottleneck. miRNA loss during extraction is common and can skew quantification. Their low abundance makes it difficult to obtain sufficient input material for downstream qRT-PCR, especially when isolating EVs from limited biological fluids like plasma or serum. Furthermore, there were limited datasets available for validation wherein the EV-miRNAs were studied in women with RPL. Most of the studies have been conducted with decidual, trophoblast tissue and plasma samples. In the present study, we focused on the change in EV-miRNAs expression especially in first trimester of pregnancy; however, miRNA expression changes as the pregnancy reaches term; thus, these results may not be applicable for higher gestational age. Additionally, for the elucidation of exact etiology of RPL pathogenesis, the miRNA–mRNA signaling pathways and genes involved need to validated experimentally. Despite all these limitations, the major strength of this study was the stringent criteria for the selection of idiopathic cases of pregnant RPL women and gestational-age-matched controls. In addition, this study suggests a non-invasive method for early diagnosis of RPL, so that proper treatment measures or alternative assisted techniques could be utilized for a successful pregnancy in such patients.

## 6 Conclusion

Results from the present study indicate that in a clinical setup, early diagnosis of RPL could be done by studying the expression profiling of EV-miRNAs unique to RPL (hsa-let-7e, miR-155, miR-139, miR-26a, miR-374a, miR-204, miR-190a, and miR-504), which give the clues of altered downstream pathways associated with immune regulation (Th1/Th2/Th17 differentiation, TNF-α signaling, and IL-17 signaling pathway) and biological/molecular mechanisms (NF-κB, TGFβ, FoxO, and VEGF) associated with pregnancy and affect the uterine microenvironment. This may aid in clarifying the unknown underlying mechanisms of RPL and the development of novel molecular therapeutic targets. Since exosomes have shown promising results in the treatment of various disorders due to the diagnostic potential and therapeutic qualities of its contents, these nanoparticles can be exploited as bio-nano-vehicle for the transportation of cargo such as drugs and small RNAs in RPL.

## Data Availability

The data presented in the study are deposited in the GEO repository, accession number GSE296460.

## Supplementary material

Supplementary Figure 1 Violin plot showing validation of differently expressed miRNAs in our dataset.

Supplementary Table 1 Excel sheet of Pathway enrichment analysis data of ClueGO and Cluepedia.
