# Supplementary material for: EV-microRNA signatures in pregnant women with idiopathic recurrent pregnancy loss: deciphering microRNAome pathway networks at feto-maternal interface
Source: Front Immunol. 2025 May 12;16:1578738. doi: 10.3389/fimmu.2025.1578738 (PMC12105548; doi:10.3389/fimmu.2025.1578738)
Supplement: Supplementary Table 1 — Excel sheet of Pathway enrichment analysis data of ClueGO and Cluepedia. [file DataSheet1.docx]

**Supplementary Table 1: List of significantly differentially expression miRNAs, their accession numbers and sequence**

| **microRNA name** | **miRBase accession** | **microRNA mature sequence** |
| --- | --- | --- |
| hsa-let-7e-5p | MIMAT0000066 | UGAGGUAGGAGGUUGUAUAGUU |
| hsa-miR-1228-5p | MIMAT0005582 | GUGGGCGGGGGCAGGUGUGUG |
| hsa-miR-1262 | MIMAT0005914 | AUGGGUGAAUUUGUAGAAGGAU |
| hsa-miR-139-5p | MIMAT0000250 | UCUACAGUGCACGUGUCUCCAGU |
| hsa-miR-140-5p | MIMAT0000431 | CAGUGGUUUUACCCUAUGGUAG |
| hsa-miR-149-5p | MIMAT0000450 | UCUGGCUCCGUGUCUUCACUCCC |
| hsa-miR-155-5p | MIMAT0000646 | UUAAUGCUAAUCGUGAUAGGGGUU |
| hsa-miR-187-5p | MIMAT0004561 | GGCUACAACACAGGACCCGGGC |
| hsa-miR-190a-5p | MIMAT0000458 | UGAUAUGUUUGAUAUAUUAGGU |
| hsa-miR-204-5p | MIMAT0000265 | UUCCCUUUGUCAUCCUAUGCCU |
| hsa-miR-208b-5p | MIMAT0026722 | AAGCUUUUUGCUCGAAUUAUGU |
| hsa-miR-2276-5p | MIMAT0026921 | GCCCUCUGUCACCUUGCAGACG |
| hsa-miR-26a-5p | MIMAT0000082 | UUCAAGUAAUCCAGGAUAGGCU |
| hsa-miR-3135a | MIMAT0015001 | UGCCUAGGCUGAGACUGCAGUG |
| hsa-miR-3146 | MIMAT0015018 | CAUGCUAGGAUAGAAAGAAUGG |
| hsa-miR-3177-5p | MIMAT0019215 | UGUGUACACACGUGCCAGGCGCU |
| hsa-miR-3529-5p | MIMAT0019828 | AGGUAGACUGGGAUUUGUUGUU |
| hsa-miR-3661 | MIMAT0018082 | UGACCUGGGACUCGGACAGCUG |
| hsa-miR-374a-5p | MIMAT0000727 | UUAUAAUACAACCUGAUAAGUG |
| hsa-miR-3909 | MIMAT0018183 | UGUCCUCUAGGGCCUGCAGUCU |
| hsa-miR-3922-5p | MIMAT0019227 | UCAAGGCCAGAGGUCCCACAGCA |
| hsa-miR-411-5p | MIMAT0003329 | UAGUAGACCGUAUAGCGUACG |
| hsa-miR-4433a-5p | MIMAT0020956 | CGUCCCACCCCCCACUCCUGU |
| hsa-miR-4433b-5p | MIMAT0030413 | AUGUCCCACCCCCACUCCUGU |
| hsa-miR-4440 | MIMAT0018958 | UGUCGUGGGGCUUGCUGGCUUG |
| hsa-miR-454-5p | MIMAT0003884 | ACCCUAUCAAUAUUGUCUCUGC |
| hsa-miR-4665-5p | MIMAT0019739 | CUGGGGGACGCGUGAGCGCGAGC |
| hsa-miR-504-5p | MIMAT0002875 | AGACCCUGGUCUGCACUCUAUC |
| hsa-miR-520d-5p | MIMAT0002855 | CUACAAAGGGAAGCCCUUUC |
| hsa-miR-520f-5p | MIMAT0026609 | CCUCUAAAGGGAAGCGCUUUCU |
| hsa-miR-574-5p | MIMAT0004795 | UGAGUGUGUGUGUGUGAGUGUGU |
| hsa-miR-615-5p | MIMAT0004804 | GGGGGUCCCCGGUGCUCGGAUC |
| hsa-miR-6501-5p | MIMAT0025458 | AGUUGCCAGGGCUGCCUUUGGU |
| hsa-miR-6726-5p | MIMAT0027353 | CGGGAGCUGGGGUCUGCAGGU |
| hsa-miR-6754-5p | MIMAT0027408 | CCAGGGAGGCUGGUUUGGAGGA |
| hsa-miR-7976 | MIMAT0031179 | UGCCCUGAGACUUUUGCUC |

**Supplementary Table 2: KEGG pathway for genes related with DEmiRNAs**

| **Index** | **Name** | **P-value** | **Adjusted p-value** | **Odds Ratio** | **Combined score** |
| --- | --- | --- | --- | --- | --- |
| 1 | PI3K-Akt signalling pathway | 6.313e-9 | 0.000001869 | 2.47 | 46.71 |
| 2 | MAPK signaling pathway | 1.293e-8 | 0.000001913 | 2.61 | 47.35 |
| 3 | Pathways in cancer | 1.644e-7 | 0.00001622 | 2.02 | 31.53 |
| 4 | Signaling pathways regulating pluripotency of stem cells | 7.025e-7 | 0.00005198 | 3.12 | 44.24 |
| 5 | Hippo signaling pathway | 0.000004145 | 0.0002454 | 2.76 | 34.22 |
| 6 | AGE-RAGE signaling pathway in diabetic complications | 0.000009296 | 0.0004241 | 3.31 | 38.31 |
| 7 | FoxO signaling pathway | 0.00001094 | 0.0004241 | 2.91 | 33.20 |
| 8 | TGF-beta signaling pathway | 0.00001146 | 0.0004241 | 3.37 | 38.35 |
| 9 | Chagas disease | 0.00001299 | 0.0004274 | 3.22 | 36.27 |
| 10 | Transcriptional misregulation in cancer | 0.00001923 | 0.0005065 | 2.44 | 26.49 |

**Supplementary Table 3: Top 10 hub genes**

| **Gene** | **Closeness** | **Betweenness** | **Degree** |
| --- | --- | --- | --- |
| *NFKB1* | 153.4 | 4664.108 | 53 |
| *IL6* | 142.8833 | 3365.645 | 46 |
| *JUN* | 154.75 | 5902.41 | 51 |
| *FOS* | 147.9167 | 3262.466 | 44 |
| *CXCL8* | 129.3667 | 225.3558 | 29 |
| *PTGS2* | 129.2833 | 752.4024 | 28 |
| *TGFB1* | 138.35 | 3297.559 | 40 |
| *MMP9* | 140.5667 | 1500.477 | 39 |
| *STAT1* | 135.95 | 2079.061 | 35 |
| *CD4* | 134.45 | 1005.743 | 33 |

**Supplementary Table 4: Clinical correlation of miRNA levels with clinical parameters**

| **Factors** | **Total**  **Numbers** | **hsa-miR-139-5p** | **hsa-miR-140-5p** | **hsa-let-7e-5p** | **hsa-miR-155-5p** | **hsa-miR-204-5p** | **hsa-miR-374a-5p** |
| --- | --- | --- | --- | --- | --- | --- | --- |
|  |  | **p-value** | **p-value** | **p-value** | **p-value** | **p-value** | **p-value** |
| **Maternal age** | | | | | | | |
| **≤30 years** | 3 | 0.9219 | 0.5431 | 0.5487 | 0.9876 | 0.9104 | 0.4941 |
| **>30 years** | 7 |  |  |  |  |  |  |
| **Gestational age** | | | | | | | |
| **≤8 weeks** | 6 | **0.024*** | 0.109 | 0.2012 | 0.299 | 0.321 | 0.877 |
| **>8 weeks** | 4 |  |  |  |  |  |  |
| **No. of Losses** | | | | | | | |
| **≤3 Loss** | 4 | 0.743 | 0.489 | 0.17 | 0.504 | 0.202 | 0.442 |
| **3-6 Loss** | 4 |  |  |  |  |  |  |
| **>6 loss** | 2 |  |  |  |  |  |  |

*Significant p-value
